# Carbon Dioxide Capture and Functionalization from a Molecular Ti(III) Oxo Anion

**DOI:** 10.1002/anie.202511532

**Published:** 2025-08-25

**Authors:** Samuel S. Veroneau, Jacob S. Mohar, Mrinal Bhunia, Hannah Farber, Alexander L. Laughlin, Robert W. Voland, Alexandra Bacon, Michael R. Gau, Kyle M. Lancaster, Daniel J. Mindiola

**Affiliations:** ^1^ Department of Chemistry University of Pennsylvania 231 S. 34^th^ Street Philadelphia PA 19104 USA; ^2^ Department of Chemistry and Chemical Biology Cornell University 259 E Ave Ithaca NY 14850 USA

**Keywords:** Carbon capture, Carbon functionalization, EPR, Metal carbonate, Oxide, Titanium

## Abstract

Carbon dioxide capture and functionalization sequesters carbon dioxide in more robust products and offers a viable route to reducing greenhouse gas emissions. We present herein a unique molecular Ti^III^ oxo anion that reversibly binds CO_2_ to allow both its sequestration and functionalization. The reduction of [(PN)_2_Ti═O] (**1**) [PN = (2‐P^i*i*^Pr_2_‐4‐methylphenyl)(mesityl)amide] with KC_8_ and 2.2.2‐cryptand (crypt) resulted in formation of [K(crypt)][(PN)_2_Ti═O] (**2**), which was fully characterized and shown to contain a Ti‐centered radical. Complex **2** reacts with Al(CH_3_)_3_ to form [K(crypt)][(PN)_2_Ti{O(Al(CH_3_)_3_}] (**3**), which can be independently prepared from [K(crypt)][(PN)_2_Ti(OCP)] and Al(CH_3_)_3_. Whereas **1** does not react with CO_2_, **2** rapidly captures the gas (1 atm, 25 °C) to produce a Ti^III^ carbonate [K(crypt)][(PN)_2_Ti(κ^2^‐O_2_C═O)] (**4**). Chemical and electrochemical oxidation of **4** releases CO_2_ to regenerate **1** while a soluble organic carbonate [Me_3_SiOC(O)OSiMe_3_, Me = CH_3_] is obtained from reaction of **4** with ClSiMe_3_.

## Graphical abstract

In their communication, Mindiola and coworkers demonstrate how a reduced Ti complex can reversibly bind CO_2_ to produce organic and inorganic carbonates. They access this chemistry through a unique Ti^III^ oxo anion that can be generated both chemically and electrochemically. The subtle electronic and structure parameters of this species promote its reactivity and establish an exciting route to the capture, functionalization, and release of CO_2_.

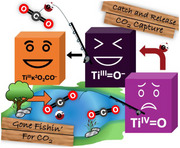

Carbon dioxide (CO_2_) is an increasingly abundant greenhouse gas^[^ ^1^, ^2^, ^3^ ^]^ that contributes greatly to climate change.^[^ ^4^, ^5^ ^]^ While progress has been made toward reducing CO_2_ emissions^[^ ^6^ ^]^ the capture, storage, and conversion of CO_2_^[^ ^7^, ^8^ ^]^ remains a priority for energy science.^[^ ^9^, ^10^, ^11^, ^12^ ^]^ Hybrid sorbents,^[^ ^13^, ^14^, ^15^ ^]^ electrochemical,^[^ ^16^, ^17^, ^18^, ^19^, ^20^, ^21^, ^22^, ^23^, ^24^, ^25^, ^26^, ^27^, ^28^ ^]^ biological,^[^ ^29^, ^30^, ^31^, ^32^ ^]^ and photocatalytic methods^[^ ^33^ ^]^ have been designed to capture and functionalize CO_2_ from both point sources (e.g., flue gas)^[^ ^34^, ^35^, ^36^, ^37^, ^38^, ^39^, ^40^, ^41^ ^]^ and directly from air.^[^ ^42^, ^43^, ^44^, ^45^, ^46^, ^47^ ^]^ Among these are metal oxides (M_x_O_y_) which play a key role in capturing CO_2_ through the process of carbon mineralization. Here, metal oxides are converted into metal carbonates [i.e., M═O to M(CO_3_)] by reaction with CO_2_ as demonstrated in both natural^[^ ^43^, ^48^, ^49^ ^]^ and artificial materials.^[^ ^33^, ^45^, ^50^, ^51^, ^52^, ^53^, ^54^, ^55^, ^56^, ^57^, ^58^, ^59^, ^60^, ^61^ ^]^ Titanium oxides are of specific interest due to their high abundance, low cost, and low toxicity.^[^ ^62^, ^63^, ^64^, ^65^, ^66^ ^]^ In contrast to the abundance of heterogeneous titanium oxides, isolable,^[^ ^67^ ^]^ mononuclear, and molecular titanium oxides (i.e., Ti═O species) are confined to high valent systems.^[^ ^68^, ^69^, ^70^, ^71^, ^72^, ^73^, ^74^, ^75^ ^]^ These molecular complexes are relatively inert^[^ ^68^, ^76^, ^77^, ^78^, ^79^ ^]^ and CO_2_ capture with them is quite rare (Figure 1a).^[^ ^80^, ^81^, ^82^, ^83^, ^84^, ^85^ ^]^ We demonstrate now the synthesis and characterization of a unique mononuclear Ti^III^ oxo radical anion and establish its reactivity with CO_2_ to generate a Ti^III^ κ^2^‐carbonate anion. Furthermore, the chemical and electrochemical one‐electron oxidation of this carbonate species extrudes CO_2_ while functionalization with ClSiMe_3_ can release a soluble organic carbonate (Figure 1b).

**Figure 1:**
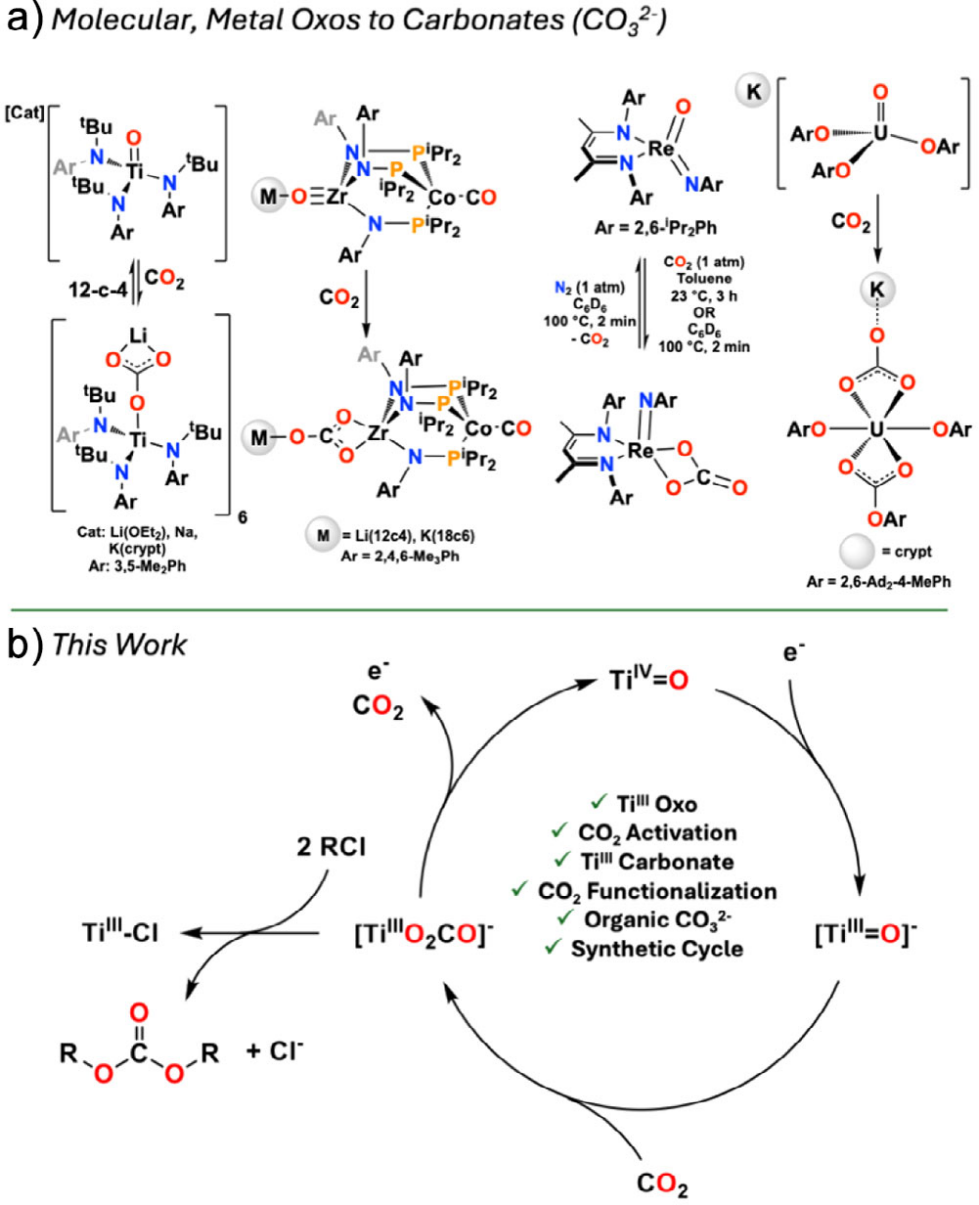
a) Reactions of molecular, metal oxo complexes with CO_2_ to form inorganic carbonates. b) Our study showing a redox mediated carbon capture and functionalization from a terminal Ti═O complex.

Reduction of (PN)_2_Ti═O (**1**)^[^ ^86^ ^]^ in toluene with KC_8_ and 2.2.2‐cryptand (Scheme 1) forms a dark purple precipitate identified as [K(crypt)][(PN)_2_Ti═O] (**2**) [crypt = 2.2.2‐cryptand; PN = (2‐P*^i^* Pr_2_‐4‐methylphenyl)(mesityl)amide] in 91% yield, as resolved through a combination of single crystal X‐ray diffraction (scXRD) and ^1^H‐NMR spectroscopy. While **1** is readily soluble in Et_2_O and toluene, **2** is only soluble in THF. scXRD reveals a slightly increased Ti─O bond length from 1.664(2) Å in **1** to 1.690(1) Å in **2**.^[^ ^86^ ^]^ This elongation is not observed in similarly reduced Ti═O systems supported by redox‐active phthalocyanine ligands, where the Ti─O bond length is essentially unchanged [∼0.003(7) Å].^[^ ^68^, ^69^, ^70^, ^71^, ^72^, ^87^ ^]^ This observation hints to reduction of **1** being metal centered rather than on the supporting ligand. In addition, the Ti─O bond length in **2** is slightly shorter than that of the Ti═O anion [Li(OEt_2_)][(NRAr)_3_Ti═O] [1.712(2) Å, R═*^t^* Bu, Ar = 3,5‐Me_2_C_6_H_3_]^[^ ^84^ ^]^ and longer than those in neutral systems^[^ ^73^, ^88^, ^89^ ^]^ such as 5‐coordinate (PNP)Ti═O(OAr) (1.656(2) Å, PNP^−^ = N‐[2‐(P*^i^* Pr_2_)_2_–4‐methylphenyl]_2_, ArO^−^ = 2,6‐ bis(diphenylmethyl)‐4‐*^t^* Bu‐phenoxide),^[^ ^75^ ^]^ (Tp^3‐tBu,5‐Me^)Ti≡O(Cl) (1.621(1) Å, Tp^3‐tBu,5‐Me^ = hydridotris(3‐*^t^* Bu‐5‐methylpyrazol‐1‐yl)borate),^[^ ^74^ ^]^ and (ArO)_2_(Py‐4‐pyr)_2_Ti═O (1.657(6) Å, ArO = 2,6‐*^i^* Pr_2_C_6_H_3_, Py‐4‐pyr = 4‐pyrrolidinopyridine).^[^ ^90^ ^]^

**Scheme 1:**
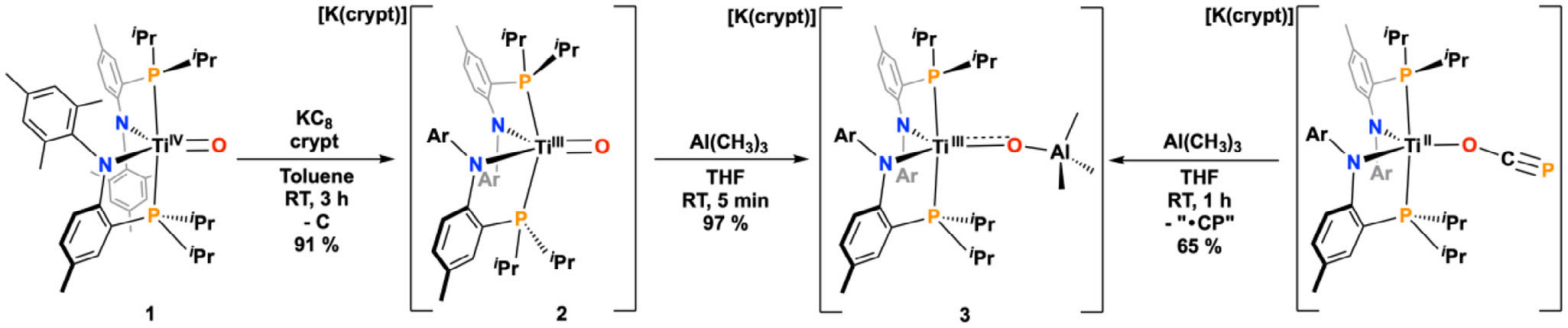
Synthetic scheme illustrating the formation of complex **2**, as well as the independent syntheses of **3** from **2** and a Ti^II^–OCP precursor.

Complex **2** instantly reacts with AlMe_3_ (excess) in THF to form bright green [K(crypt)][(PN)_2_Ti{O(Al(CH_3_)_3_}] (**3**) in near quantitative yield. scXRD of **3** revealed a Ti─O bond length of 1.764(3) Å, a significant elongation from **1** and **2** (∼0.1 and 0.05 Å, respectively) and similar to other Ti^IV^═O systems including [Li(OEt_2_)][(NRAr)_3_Ti═O] (vide supra).^[^ ^84^, ^86^ ^]^ Based on structural data and the Pauling bond length equation,^[^ ^91^, ^92^ ^]^ utilizing the Schomaker–Stevenson correction^[^ ^93^ ^]^ and the covalent radii derived from Pyykkö,^[^ ^94^, ^95^, ^96^, ^97^ ^]^ we predict single, double, and triple bond lengths of 1.838, 1.588, and 1.458 Å, respectively. We therefore assign compounds **1** and **2** to have intermediate single to double bond character [Ti═O bond lengths 1.664(2) and 1.690(1) Å, respectively] whereas compound **3** has more single bond character [Ti─O bond length 1.764(3) Å]. Compound **3** can alternatively also be prepared in 65% yield from the previously published Ti^II^ complex [K(crypt)][(PN)_2_Ti(OCP)]^[^ ^98^ ^]^ upon reaction with AlMe_3_ through what we speculate is [CP]**^•^** loss.^[^ ^99^, ^100^ ^]^

The electronic structures of **2** and **3** were subsequently investigated and solution state magnetic moment measurements of **2** and **3** (Evans’ method,^[^ ^101^, ^102^ ^]^ THF‐*d*_8_, 300 K) revealed a *µ*_eff_ = 1.98 and 1.94 µ_B_, respectively, consistent with *S* = ½, d^1^ systems.^[^ ^103^ ^]^ UV–vis spectral data revealed features at 682 nm (253 M^−1^·cm^−1^) and 654 nm (92 M^−1^·cm^−1^) for **2** and **3**, respectively, which we assign as d–d transitions and are consistent with the other metal–ligand multiple bond complex of Ti^III^ such as [K(crypt)][(PN)_2_Ti═PSiMe_3_] that exhibits a feature at 740 nm (545 M^−1^·cm^−1^).^[^ ^98^ ^]^ Further evidence for the presence of Ti^III^ in **2** and **3** is provided by X‐band EPR spectroscopy. EPR spectra recorded for **2** at 100 K in a 1:1 toluene:THF (v/v) revealed axial symmetry with *g*_x_ ≈ *g*_y_ > *g*_z_ (1.99, 1.98, and 1.95) consistent with a Ti^III^ centered radical. As *g*_┴_ is larger than *g*_‖_, multiple bond character is invoked on the basis of previous studies with the isoelectronic vanadyl [V═O]^2+^.^[^ ^104^, ^105^ ^]^ Similar values for *g*_┴_ and *g*_‖_ were found in the X‐band EPR spectrum (100 K, 9.317 GHz) in 1:1 toluene:THF (v/v) of **3** (1.99, 1.97, and 1.95) indicating again a Ti^III^ centered radical populating an orbital of rhombic symmetry (*g*_x_ > *g*_y_ > *g*_z_). Spectra also revealed superhyperfine coupling (shc) to 2 P atoms (Figure 2).^[^ ^70^, ^71^, ^72^, ^87^ ^]^ In contrast, *g*_iso_ = 2.0003–2.0018 are observed at this temperature for the radical anionic phthalocyanine^3−^ Ti^IV^═O systems, which essentially do not deviate from the metal‐free ligand radical anion [2.0034 (0.18)].^[^ ^84^ ^]^

**Figure 2:**
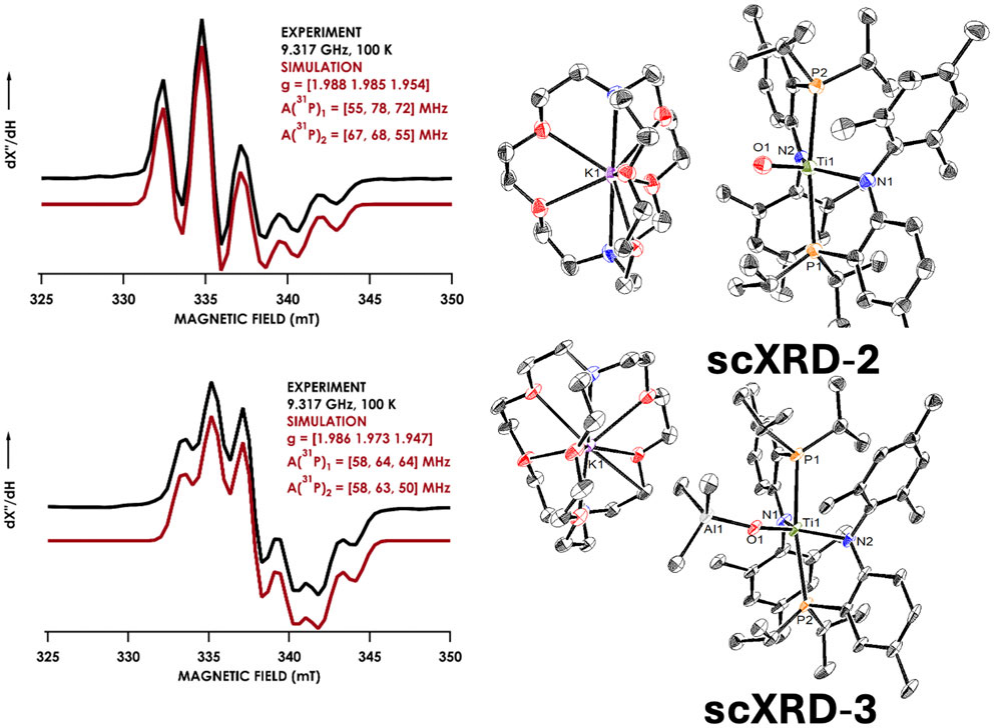
ORTEP‐III 50% probability thermal ellipsoid plot of **2** and **3**. X‐band EPR at 100 K of **2** and **3** with *g*_x,y,z_‐values of 1.99, 1.98, 1.95 (**2**) and 1.99, 1.97, 1.95 (**3**).

To explore the viability of these complexes for carbon capture and functionalization, we exposed **2** to 1 atm of CO_2_ in THF at 25 °C resulting in an immediate color change from dark purple to bright orange.^[^ ^106^ ^]^ The resulting orange material was sparingly soluble in Et_2_O and identified as [K(crypt)][(PN)_2_Ti(κ^2^O_2_C═O)] (**4**) through a combination of scXRD, ^1^H‐NMR, and IR spectroscopies (Figure 3a). Complex **4** crystallized in the orthorhombic and noncentrosymmetric space group Pca2_1_, which revealed two crystallographically independent enantiomers in the asymmetric unit, and Ti─O bond lengths of 2.004(3) and 2.094(3) Å and 2.031(3) and 2.067(3) Å in enantiomers **4_A_** and **4_B_**, respectively. These distances are significantly longer than the κ^1^‐Ti^IV^‐carbonate anion system [(R[Ar[N)_3_Ti(OCO_2_Li)]_6_ (Ti─O = 1.849(2) Å),^[^ ^81^, ^84^, ^85^, ^107^ ^]^ which is unsurprising given the increased electron density on the Ti in **4** and the κ^2^ coordination of CO_3_^2−^. The C─O bonds in **4** are 1.327(5), 1.311(5), 1.231(5) Å for molecule **4_A_** and 1.329(5), 1.310(5), and 1.234(5) Å for molecule **4_B_**, consistent with the C─O bond distances previously reported by Thomas, Meyer, Cummins, and Arnold.^[^ ^81^, ^84^, ^85^, ^107^ ^]^ Similar to the other κ^4^ carbonate systems published (Zr, Re, U, and Mo), the summation of the ∠O─C─O bond angles for molecules **4_A_** and **4_B_**, demonstrate a planar C‐atom.^[^ ^80^, ^81^, ^82^, ^84^ ^]^ Further evidence for the CO_3_^2−^ stems from the IR spectrum which revealed a sharp νC═O stretch^[^ ^108^ ^]^ at 1628 cm^−1^ consistent with previous reported values of 1590 cm^−1^ (Ti),^[^ ^84^ ^]^ 1642 cm^−1^ (Zr),^[^ ^81^ ^]^ 1731 cm^−1^ (Re),^[^ ^82^ ^]^ 1628 cm^−1^ (Mo).^[^ ^108^ ^]^ Assignment of this stretch as CO_3_^2−^ was additionally demonstrated by ^13^C isotopic labeling using ^13^CO_2_ to generate the isotopomer **4–^13^C**.^[^ ^109^ ^]^ Complex **4–^13^C** revealed a ν^13^C═O at 1589 cm^−1^ in good agreement with the theoretical isotopic shift from **4** using the harmonic oscillator (theoretically predicted ν^13^CO = 1592 cm^−1^). Complex **4** also revealed a solution state magnetic moment (Evans’ method, C_6_D_6_, 300 K) of *µ*_eff_ = 1.87 μ_B_ in good agreement with an *S* = ½, d^1^ system,^[^ ^110^ ^]^ and the electronic absorption spectrum revealed a d–d transition 755 nm (38 M^−1^·cm^−1^).^[^ ^82^, ^84^ ^]^ A significant bathochromic shift is observed as compared to other {(PN)_2_Ti}^+^ compounds likely due to the geometry change from 5 to 6 coordinate.^[^ ^98^ ^]^ The electronic structure of **4** was finally resolved with X‐band EPR recorded at 100 K in a 1:1 toluene:THF (v/v). Spectra revealed rhombic symmetry with *g*_y_ > *g*_x_ > *g*_z_ (1.96, 1.99, and 1.90) and shc to 2 P atoms (Figure 3b). Spin Hamiltonian parameters for **4** were calculated via density functional theory (DFT) as implemented in ORCA 6.0 to corroborate these results.^[^ ^111^ ^]^

**Figure 3:**
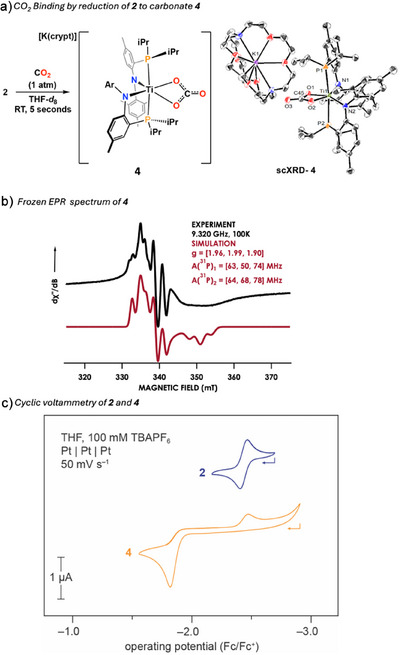
a) Reaction of **2** with 1 atm of CO_2_ resulting in **4**. ORTEP‐III 50% probability thermal ellipsoid plot of one of the two molecules of **4** in unit cell. b) X‐band EPR at 100 K of **4** with g_x,y,z_‐values of 1.96, 1.99, 1.90. The experimental spectrum is in black. A simulation of the spectrum carried out using EasySpin is in red. c) Cyclic voltammetry of **2** and **4** in THF demonstrating reversible and irreversible electrochemistries, respectively.

Previously reported Ti and Re oxo complexes (Figure 1a) show reversible binding of CO_2_ in presence of coordinating solvents or 12‐crown‐4 at room temperature and heating at 100 °C for 2 min, respectively. Complex **1** interestingly demonstrated no reaction with CO_2_ even upon heating to 70 °C in THF‐*d*_8_ for 18 h.^[^ ^112^ ^]^ Complex **4** furthermore did not extrude CO_2_ upon heating, decomposing to unidentified products at 85 °C in THF‐*d*_8_ for 12 h under vacuum.^[^ ^113^ ^]^ Cyclic voltammetry (CV) was thus employed to explore the electrochemical release of CO_2_ from **4** (Figure 3c).^[^ ^114^ ^]^ CV of **2** exhibits a single reversible wave around −2.35 V versus Fc/Fc^+^ (Fc = ferrocene, Fc^+^ = ferrocenium) corresponding to the Ti^III^/Ti^IV^ redox couple, justifying the use of strong reductants (e.g., KC_8_) in generating **2** from **1**. Conversely, **4** exhibits two *irreversible* features at around −1.8 and −2.35 V versus Fc/Fc^+^. The irreversible oxidation at −1.8 V versus Fc/Fc^+^ is consistent with the release of CO_2_ from **4** upon oxidation in an electrochemical–chemical mechanism that does not become more reversible at higher scan rates (i.e., 300 mV s^−1^).^[^ ^114^ ^]^ The corresponding reductive event observed at −2.35 V versus Fc/Fc^+^ aligns with the Ti^III^/Ti^IV^ redox couple of **2**, being irreversible here likely owing to dissolved CO_2_ chemically reacting to reform **4**. To quantify the electrochemical release of CO_2_, complex **4** was oxidized chemically with 0.5 eq. 1,2‐diiodoethane forming ethylene, CO_2_, and **1** as demonstrated by ^1^H, ^31^P{^1^H}, and ^13^C NMR spectra (Figure 4a).^[^ ^115^ ^]^ Functionalization of CO_2_ could finally be achieved with **4** through reaction with 2 equivalents (or excess) trimethylsilyl chloride [ClSi(CH_3_)_3_], generating bis(trimethylsilyl)carbonate [Me_3_SiOC(O)OSiMe_3_]^[^ ^84^, ^116^ ^]^ and [(PN)_2_TiCl]^[^ ^117^ ^]^ (Figure 4a).^[^ ^81^ ^]^ This formation of Me_3_SiOC(O)OSiMe_3_ is accompanied by partial loss of CO_2_ (*δ* 125.4, THF‐*d*_8_, 300 K, using **4–^13^C**)^[^ ^118^ ^]^ and an unidentified Si‐containing product identified by ^13^C and ^29^Si‐IENPT NMR spectroscopies, respectively, motivating future work into CO_2_ functionalization on this anionic platform.^[^ ^119^, ^120^ ^]^

**Figure 4:**
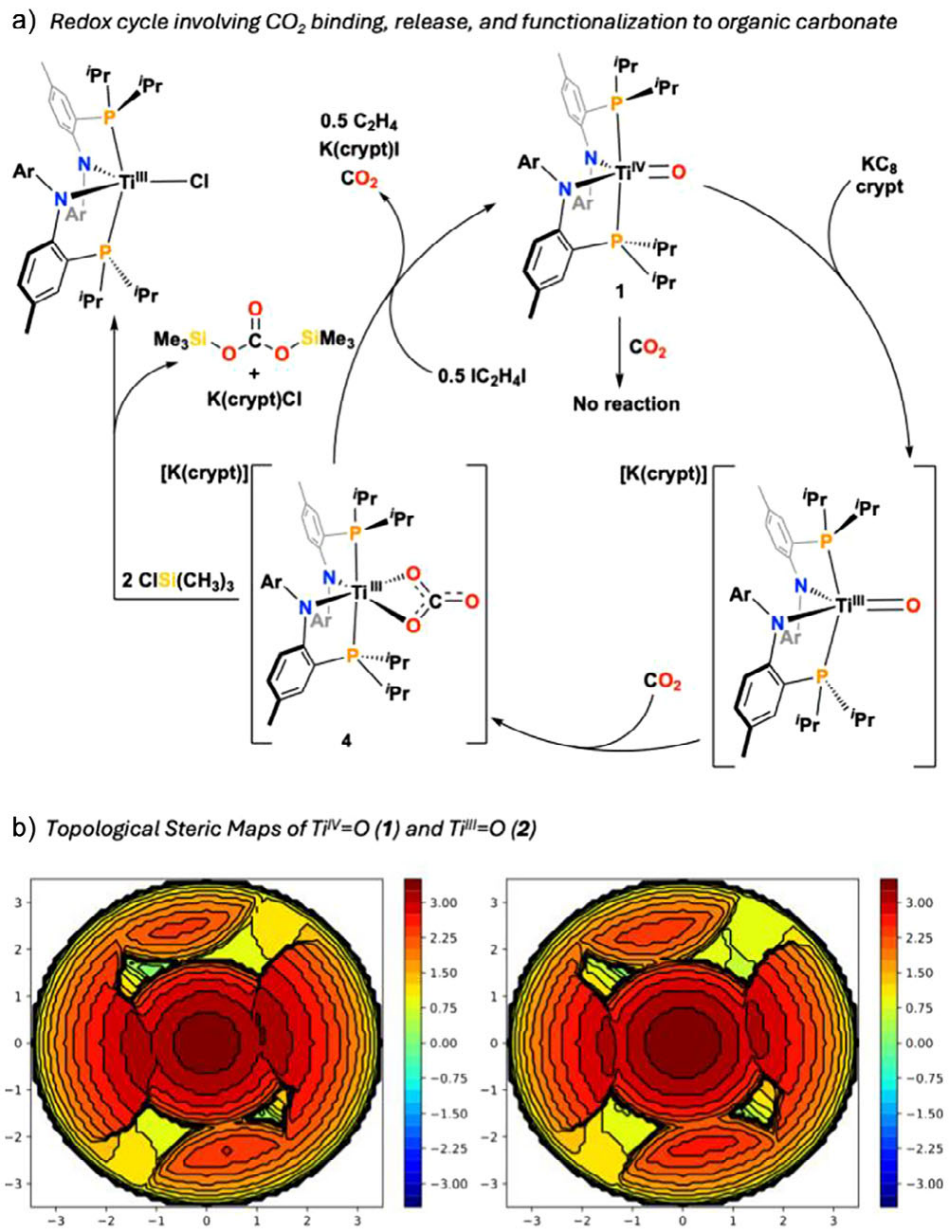
a) Stoichiometric reactivity of **4** releasing CO_2_ upon oxidation regenerating **1** and functionalization towards organic carbonates. b) Topographic maps generated by SambVca 2.1 for **1** and **2** depicting steric hinderance around Ti center.

Cumulatively, a reduction event must occur to form **2** from **1** prior to CO_2_ capture. Such redox induced CO_2_ capture is unique among molecular metal oxos and mimics organic electrochemical CO_2_ capture and concentration systems.^[^ ^41^, ^121^ ^]^ CO_2_ binding on transition metal oxo complexes may proceed through nucleophilic attack by the oxygen.^[^ ^84^ ^]^ Consistent with this, reduction of **1** generates a stronger nucleophile in anionic **2** that reacts with CO_2_. Still, the κ^2^ coordination of CO_3_^2−^ and Ti‐centered radical in **2** suggests a separate pathway may be involved, wherein CO_2_ interacts with the metal center directly. Such reactivity would effectively be a [2 + 2] cycloaddition of CO_2_ and reminiscent of the [2 + 2] cycloadditions of C═O bonds to M═O bonds in oxo rhenium complexes first reported by Hermann.^[^ ^122^, ^123^ ^]^ Steric mapping of **1** and **2** with SambVca^[^ ^119^ ^,^ ^120^ ^]^ reveals an increase in free volume about the Ti center upon reduction (Figure 4b); this calculation is supported by observed structural metrics between **1** and **2** such as the ∠P─Ti─P bond angle which contracts from 179.08(4)^[^ ^86^ ^]^ in **1** to 170.52(3)° in **2**. In addition, there is significant increase in the through‐space angle of the *^i^* Pr groups of the PN ligand and oxo oxygen atom from **1** and **2** of ∠*^i^* PrC‐O‐C*^i^* Pr 140.6^[^ ^16^, ^17^, ^18^, ^19^, ^20^, ^21^, ^22^, ^23^, ^24^, ^25^, ^26^, ^27^, ^28^, ^41^ ^]^ and 152.4°, respectively.^[^ ^124^ ^]^ Structural rearrangement is noticeable when **1** is converted to **2** (Figure 4b), but we argue that this structural change is not the main driving force behind CO_2_ capture by **2**. Future studies will probe the role of charge and geometry in CO_2_ capture with this system.

We have demonstrated the synthesis and structural characterization of the first mononuclear Ti^III^ oxo anion (**2**) and its ability to react with electrophiles such as Lewis acids (**3**) and CO_2_ (**4**). We demonstrate how carbon capture and functionalization can be promoted with a transition metal complex ion containing an unpaired electron derived from a metal oxo unit. Importantly, this reactivity is gated by a one‐electron reduction to produce a slightly more sterically accessible but anionic Ti═O moiety. Although a strong reductant is needed, chemical and electrochemical oxidation of **4** releases CO_2_ quantitatively, establishing the potential of this system for CO_2_ capture‐and‐concentration. Furthermore, functionalization of a soluble carbonate derived from CO_2_ is achieved with trimethylsilyl chloride, suggesting **4** might serve as a versatile platform for CO_2_ conversion.^[^ ^125^, ^126^, ^127^, ^128^, ^129^, ^130^, ^131^ ^]^

## Conflict of Interests

The authors declare no conflict of interest.

## Supporting information



Supporting Information

Supporting Information

Supporting Information

## Data Availability

The data that support the findings of this study are available in the Supporting Information of this article.
