# Supplementary material for: Carbon Dioxide Capture and Functionalization from a Molecular Ti(III) Oxo Anion
Source: Angew Chem Int Ed Engl. 2025 Aug 25;64(40):e202511532. doi: 10.1002/anie.202511532 (PMC12462757; doi:10.1002/anie.202511532)
Supplement: Supplementary file 1 — Supporting Information [file ANIE-64-e202511532-s003.docx]

Supporting Information
©Wiley-VCH 2025
69451 Weinheim, Germany

**Carbon dioxide capture and functionalization from a molecular Ti(III) oxo anion**

Samuel S. Veroneau,^[a]^ Jacob S. Mohar,^[a]^ Mrinal Bhunia,^[a]^ Hannah Farber,^[a]^ Alexander Laughlin,^[b]^ Robert W. Voland,^[b]^ Alexandra Bacon,^[a]^ Michael R. Gau,^[a]^ Kyle M. Lancaster,^[b]^ and Daniel J. Mindiola*^,[a]^

^[a]^ Department of Chemistry, University of Pennsylvania, 231 S. 34^th^ St., Philadelphia, PA, 19104, USA

^[b]^ Department of Chemistry and Chemical Biology, 259 E Ave, Cornell University, Ithica, NY, 14850, USA

*Corresponding Author: mindiola@sas.upenn.edu

**Table of Contents**

1. Methods and MaterialsS4

1.1 General ConsiderationsS4

1.2 Nuclear Magnetic Resonance Spectroscopy (NMR)S4

1.3 Solution State Magnetic Susceptibility S4

1.4 Infrared (IR) Spectroscopy S5

1.5 Ultra-Violet-Visible (UV-Vis) Absorption Spectroscopy S5

1.6 Electron Paramagnetic Resonance Spectroscopy (EPR) S5

1.7 Single Crystal X-ray Diffraction (XRD) S5

2. SynthesisS6

2.1 [K(crypt)][(PN)_2_Ti=O], **2** S6

2.2 [K(crypt)][(PN)_2_Ti{O(AlMe_3_)}], **3**S6

2.3 [K(crypt)][(PN)_2_Ti(κ^2^-O_2_C=O)], **4**S7

S2.3.1 Synthesis of [K(crypt)][(PN)_2_Ti(κ^2^-O_2_^13^C=O)], **4-^13^C**S7

S2.3.2 Video of synthesis of **4** from **2**S7

3. NMR SpectroscopyS8

3.1 NMR Spectroscopy of [K(crypt)][(PN)_2_Ti=O], 2 S8

S3.1.1 ^1^H NMR spectrum of **2** S8

3.2 NMR Spectroscopy of [K(crypt)][(PN)_2_Ti{O(AlMe_3_)}], 3 S9

S3.2.1 ^1^H NMR spectrum of **3** S9

3.3 NMR Spectroscopy of [K(crypt)][(PN)_2_Ti(κ^2^-O_2_C=O)], 4 S10

S3.3.1 ^1^H NMR spectrum of **4** S10

S3.3.2 Reaction ^1^H NMR spectrum of **2** + CO_2_ S11

S3.3.3 Reaction ^31^P{^1^H} NMR spectrum of **2** + CO_2_ S12

S3.3.4 ^1^H NMR spectrum of **4** + excess ClSiMe_3_ S13

S3.3.5 ^1^H NMR spectrum of **4-^13^C** + 2 ClSiMe_3_ S14

S3.3.6 ^29^Si INEPT NMR spectrum of **4-^13^C** + 2 ClSiMe_3_ S15

S3.3.7 ^13^C{^1^H} NMR spectrum of **4-^13^C** + 2 ClSiMe_3_ S16

S3.3.8 ^1^H ^13^C{^1^H} HSQC NMR of **4-^13^C** + 2 ClSiMe_3_ S17

S3.3.9 ^1^H NMR spectrum of **4** + 1,2-diiodoethane S18

S3.3.10 ^1^H NMR study of the irreversibility of CO_2_ binding in **4**  S19

S3.3.11 ^31^P{^1^H} NMR spectrum of **4** after heating 12 h at 85 °CS20

S3.3.12 ^1^H NMR study of CO_2_ binding in **1**  S21

S3.3.13 ^31^P{^1^H} NMR study of CO_2_ binding in **1**  S22

**4. IR Spectroscopy** S23

4.1 IR Spectroscopy of [K(crypt)][(PN)_2_Ti=O], 2 S23

S4.1.1 IR spectrum of **2** S23

4.2 IR Spectroscopy of [K(crypt)][(PN)_2_Ti(κ^2^-O_2_C=O)], 4 S24

S4.2.1 IR spectrum of **4** S24

S4.2.2 IR spectrum of **4-^13^C** S24

S4.2.3 Overlay of S4.2.1 and S4.4.2 S25

S4.2.4 Zoomed in S4.2.3 on ν^12/13^C=O shiftS25

**5. UV-Vis Absorbance Spectroscopy** S26

5.1 UV-Vis of [K(crypt)][(PN)_2_Ti=O], 2 S26

S5.1.1 UV-Vis of **2** S26

S5.1.2 UV-Vis of **2** zoomed in for d-d transitionsS26

S5.1.3 UV-Vis of **2** zoomed in for π-π* transitionsS28

5.2 UV-Vis of [K(crypt)][(PN)_2_Ti{O(AlMe_3_)}, 3 S28

S5.2.1 UV-Vis of **3** S28

S5.2.2 UV-Vis of **3** zoomed in for d-d transitions S28

S5.2.3 UV-Vis of **3** zoomed in for π-π* transitions S29

5.3 UV-Vis of [K(crypt)][(PN)_2_Ti(κ^2^-O_2_C=O)], 4 S30

S5.3.1 UV-Vis of **4** S30

S5.3.2 UV-Vis of **4** zoomed in for d-d transitions S30

S5.3.3 UV-Vis of **4** zoomed in for π-π* transitions S31

**6. EPR Spectroscopy** S32

7. ElectrochemistryS33

S7.1 Cyclic voltammetry of **2**S33

S7.2 Cyclic voltammetry of **4**S33

8. Single Crystal X-ray Diffraction StudiesS34

S7.1 Table of crystallographic parameters for selected compoundsS34

S7.2 Structural comparison of complexes 1 and 2S35

9. ReferencesS36

**1. Materials and Methods**

**1.1 *General Considerations***

All operations were performed in M. Braun gloves boxes (nitrogen atmosphere) or using standard Schlenk techniques. Pentane (Fisher Scientific), hexanes (Fisher Scientific), toluene (Fisher Scientific), tetrahydrofuran (THF, Fisher Scientific), benzene (Fisher Scientific), and diethyl ether (Et_2_O, Fisher Scientific) were purchased from commercial vendors, thoroughly bubbled with argon, made anhydrous by passage through columns of activated alumina in a Grubbs-type solvent system, and stored over sodium metal and 4 Å molecular sieves prior to use. Benzene-*d_6_* (Cambridge Isotope Laboratories) and THF-*d*_8_ (Cambridge Isotope Laboratories) were stored over potassium mirror overnight, sublimed/distilled by trap-to-trap transfer *in vacuo*, and degassed by minimum three freeze-pump-thaw cycles prior to use. Celite and 4 Å molecular sieves were dried *in vacuo* overnight at 200 °C.

[TiCl_3_(THF)_3_],^[1]^ LiPN,^[2]^ (PN)_2_TiCl,^[3]^ [K(crypt)][(PN)_2_TiCl],^[4]^ [K(crypt)][(PN)_2_Ti(OCP)],^[5]^ [(PN)_2_Ti=O],^[6]^ and potassium graphite (KC_8_)^[7]^ were prepared according to reported procedures.

2.2.2-cryptand (crypt, Sigma Aldrich), CO_2_ (Airgas- CD I200), ^13^CO_2_ (Cambridge Isotope Laboratories), trimethylsilylchloride (ClSiMe_3_, Sigma Aldrich), and trimethyl aluminium (AlMe_3_, Sigma, Aldrich, 2 M in hexanes) were purchased from the listed sources. 2.2.2-cryptand was dried at 85 °C overnight *in vacuo* before being crystallized from a concentrated THF solution layered beneath pentane at –35 °C prior to use. ClSiMe_3_ was refluxed over CaH_2_ overnight, distilled *in vacuo*, degassed by a minimum of three freeze-pump-thaw cycles, and stored over molecular sieves prior to use. Gases and AlMe_3_ were used as received.

**1.2 *Nuclear Magnetic Resonance Spectroscopy (NMR)***

NMR spectroscopic studies were carried out using Bruker 400 MHz or 500 MHz spectrometers equipped with J. Young NMR tubes at room temperature (300 K). ^1^H and ^13^C NMR chemical shifts are referenced to the residual solvent signals (C_6_D_6_: ^1^H: 7.16 ppm, ^13^C: 128.06 ppm, THF-*d*_8_: ^1^H: 3.58 ppm, ^13^C: 67.57 ppm). ^31^P{^1^H} and ^29^Si-INEPT chemical shifts are referenced using ^1^H spectrum as an absolute reference utilizing the IUPAC unified scale which relies on *Ξ* values expressed as percentages.^[8]^

**1.3 *Solution State Magnetic Susceptibility***

Magnetic susceptibility was measured by the Evans’ method^[9]^ in benzene-*d*_6_ or THF-*d*_8_ with the residual protio solvent peak as the internal standard and the deuterated solvent used as the external standard in a capillary. Spin only magnetic values (*μ*_eff_) were calculated using the equations shown below.^[10]^ Corrections for diamagnetism were made using tabulated Pascal constants.^[11]^

| $\frac{3000\Delta f}{4\pi F[M](FW)}=\chi_{measured}$  $\chi_{mol}=(FW)\times\chi_{measured}$  $\chi_{P-corr}=\chi_{mol}-\chi_{D}$  $\mu_{eff}=2.84\sqrt{\chi_{P-corr}T}$ | $\Delta f=change in chemical shift of internal$  $and external standard \left( Hz \right)$  $F=Instrument Frequency (Hz)$  $\left[ M \right]=concentration of compound \left( \frac{mol}{L} \right)$  $FW=molecular weight of compound \left( \frac{g}{mol} \right)$  $\chi_{D}=diamagnetic correction \left( {10}^{-6}\frac{emu}{mol} \right)$  $T=Temperature (K)$ |
| --- | --- |

Calculated Diamagnetic Corrections^[11]^

| Molecule | $\chi_{D} ({10}^{-6} emu)$ |
| --- | --- |
| [K(crypt)][(PN)_2_Ti=O], **2** | -443.22 |
| [K(crypt)][(PN)_2_Ti{O(AlMe_3_)}], **3** | -463.22 |
| [K(crypt)][(PN)_2_Ti(κ^2^-O_2_C=O)], **4** | -439.76 |

**1.4 *Infrared Spectroscopy (IR)***

IR spectroscopic studies were carried out using a Jasco FT/IR-4600 spectrometer with samples mounted between KBr windows. The sample chamber was flushed with Ar to prevent decomposition during measurements.

Isotopic shifts were calculated using the harmonic oscillator model in which the ratio of the reduced masses was used to find a multiplication factor (ratio) for the wavenumber difference between ^12^C-^16^O and ^13^C-^16^O carbonate. It was found that the ^13^C labeled carbonate should have a stretching frequency 0.978 that of the ^12^C carbonate.^[12]^

**1.5 *Ultraviolet-Visible Absorption Spectroscopy (UV-Vis)***

UV-Vis spectroscopic studies were carried out using a Cary 5000 Spectrometer equipped with 1 cm quartz cuvettes sealed with J. Young valves.

**1.6 *Electron Paramagnetic Resonance Spectroscopy (EPR)***

CW-X-band EPR spectra were obtained at the National Resource for Advanced Electron-Spin Resonance Spectroscopy (ACERT) using a Bruker Elexsys-II spectrometer maintained at 100 K by a flow cryostat (Oxford) cooled with liquid N2. Spectra were simulated using EasySpin.^[13]^

**1.7 *Single Crystal X-Ray Diffraction (XRD)***

Crystallographic studies were carried out on single crystals, which were coated with NVH oil, mounted at the end of a cryoloop, and placed in the cold stream of the diffractometer (100 K). Data were collected using a Rigaku XtaLAB Synergy-S diffractometer^[14]^ (Cu-Kα radiation λ=1.54184 Å, compounds **2,** and **4**) or a Rigaku XtaLAB Synergy-S diffractometer^[15]^ (Mo-Kα radiation λ=0.71073 Å, compound **3**) each equipped with an HPC area detector (HyPix-6000HE or Dectris Pilatus3 R 200K, respectively) and proceeded with LAB diffractometers operated through the Rigaku software.^6,7^ The intensity data for all structures were corrected for Lorentz and polarization effects and for absorption using SCALE3 ABSPACK.^[16]^ All crystal structures were solved using dual space methods – SHELXT^[17]^ and refinement was by full-matrix least squares based on F^2^ using SHELXL.^[18]^ All reflections were used during refinement for all structures. All data processing was carried out in Olex2.^[19]^ Non-hydrogen atoms were refined anisotropically and hydrogen atoms were refined using a riding model for all structures. All structures were deposited into CSD and details can be found in section 7 of the ESI.

**2. Synthesis**

2.1 *[K(crypt)][(PN)_2_Ti=O]*, **2**

Under a nitrogen atmosphere, KC_8_ (12.1 mg, 0.0895 mmol) and 2.2.2-cryptand (33.7 mg, 0.0895 mmol) were mixed in toluene turning blue and added to a stirring purple toluene solution of [(PN)_2_Ti=O]^[6]^ (60.4 mg, 0.0811 mmol) at room temperature. The suspension was allowed to stir for 3 h and then filtered over Celite. The resulting solids were washed with toluene (*ca.* 3 x 3 mL) and pentane (*ca.* 1 x 3 mL) and then extracted into THF (*ca.* 5 mL). The dark purple filtrate was then concentrated (ca. 0.5 mL), layered beneath hexanes (*ca.* 1 mL), and placed in the freezer at -35 °C overnight resulting in a dark purple microcrystalline material that can be used without further purification. X-ray quality crystals were grown from dilute (*ca.* 0.75 mL) THF and 2-3 drops of hexanes at -35 °C for 3 days. Yield: 85.8 mg, 0.0740 mmol, 91.2 %. **^1^H NMR** (500 MHz, THF-*d*_8_): δ 3.57 (Δν_1/2_ = 23.5 Hz), 2.54 (Δν_1/2_ = 14.9 Hz). **Evans’ Method** (THF-*d*_8_, 300 K): μ_eff_ = 1.98(2) μ_B_. **τ_5_**: 0.66. **UV-Vis** (THF) [λ (nm), ε (M^-1^·cm^-1^)]: 215 (71614), 249 (19909), 319 (13657), 398 (3190), 556 (1119), 563 (785), 682 (253), 944 (135).

2.2 *[K(crypt)][(PN)_2_Ti{O(AlMe_3_)}]*, **3**

Under a nitrogen atmosphere, 2 drops of AlMe_3_ (2 M in hexanes, excess) were added to a stirring purple THF solution of **2** (34.2 mg, 0.0295 mmol) at room temperature. The solution smokes and turns green almost immediately and is allowed to stir for an additional 5 minutes. All volatiles are removed *in vacuo* resulting in a green residue which was washed with pentane (*ca.* 3 x 5 mL). The resulting green product was extracted with THF (*ca.* 4 mL), filtered over Celite, and concentrated to *ca.* 0.5 mL. The green solution was then layered beneath hexanes (*ca.* 1 mL) and placed in the freezer at -35 °C overnight resulting in a dark green microcrystalline material that can be used without further purification. X-ray quality crystals were grown from dilute (*ca.* 0.75 mL) THF layered beneath hexanes (*ca.* 0.25 mL) at -35 °C overnight. Yield: 35.1 mg, 0.0285 mmol, 96.7 %. **^1^H NMR** (500 MHz, THF-*d*_8_): δ 3.54 (Δν_1/2_ = 12.7 Hz), 2.55 (Δν_1/2_ = 12.0 Hz), -1.25 (Δν_1/2_ = 137.0 Hz). **Evans’ Method** (THF-*d*_8_, 300 K): μ_eff_ = 1.94 μ_B_. **τ_5_**: 0.65; **τ_4_**: 0.98. **UV-Vis** (THF) [λ (nm), ε (M^-1^·cm^-1^)]: 217 (40945), 254 (12063), 273 (10932), 325 (10336), 388 (1780), 493 (125), 654 (92), 815 (43).

Note: Dichroic crystals of yellow and green (see below)


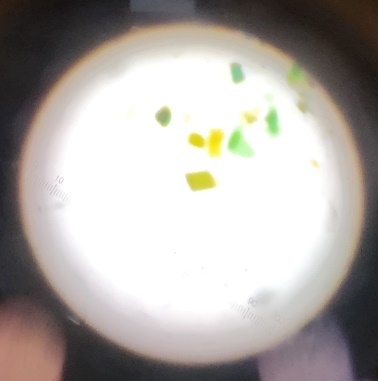


2.3 *[K(crypt)][(PN)_2_Ti(κ^2^-O_2_C=O)]*, **4**

Under a nitrogen atmosphere, **2** (35.2 mg, 0.0303 mmol) was loaded into a J-Young equipped flask or NMR tube and dissolved in THF (0.5 mL). On the Schlenk line, the reaction vessel was degassed using two freeze-pump-thaw cycles. Upon warming to room temperature, the reaction vessel was backfilled with CO_2_ for 8 seconds vented through a bubbler to ensure addition of 1 atm. The reaction vessel was then closed and inverted leading to an immediate color change from purple to bright orange. All volatiles were then removed *in vacuo* and the resulting orange residue was washed with pentane (*ca.* 3 x 2 mL). The orange residue was then extracted into THF (3 mL) which was then concentrated and layered beneath hexanes and placed in the freezer at -35 °C overnight to yield **4** as an orange microcrystalline solid which can be used without further purification. X-ray quality crystals were grown from the vapor diffusion of a dilute solution of **4** in Et_2_O into toluene at -35 °C for 3 days. Yield: 35.9 mg, 0.0298 mmol, 98.3 %. **^1^H NMR** (500 MHz, THF-*d*_8_): δ 9.59 (Δν_1/2_ = 73.6 Hz), 8.87 (Δν_1/2_ = 56.7 Hz), 7.01 (Δν_1/2_ = 131.9 Hz), 6.07 (Δν_1/2_ = 499.6 Hz), 4.41 (Δν_1/2_ = 32.9 Hz), 3.55 (Δν_1/2_ = 27.5 Hz), 3.48 (Δν_1/2_ = 18.2 Hz), 3.14 (Δν_1/2_ = 78.1 Hz), 2.49 (Δν_1/2_ = 14.7 Hz), 1.91 (Δν_1/2_ = 14.8 Hz), -3.47 (Δν_1/2_ = 299.2 Hz), -6.22 (Δν_1/2_ = 299.5 Hz). **Evans’ Method** (THF-*d*_8_, 300 K): μ_eff_ = 1.87 μ_B_. **IR** (solid, KBr, ν cm^-1^): 1628 cm^-1^. **UV-Vis** (THF) [λ (nm), ε (M^-1^·cm^-1^)]: 252 (20949), 280 (14527), 323 (12152), 388 (3381), 415 (4894), 755 (38).

2.3.1 *[K(crypt)][(PN)_2_Ti(κ^2^-O_2_^13^C=O)]*, **4-^13^C**

Synthesized identically to **4** but using ^13^C enriched CO_2_. **IR** (solid, KBr, ν cm^-1^): 1589 cm^-1^.

- - 1. *Video of the synthesis of* **4** is included in a separate file

**3. NMR Spectroscopy**

3.1 *NMR Spectroscopy of [K(crypt)][(PN)_2_Ti=O]*, **2**

**Figure S1:** Room temperature (300 K) ^1^H NMR spectrum of **2** in THF-*d*_8_ referenced to residual solvent peak.^[20]^ Paramagnetic nature of **2** precludes structural assignments, however paramagnetic resonances are marked. Absence of resonances at *ca.* δ -5 are indicative of Ti^III^ with metal-ligand multiple bond character.

3.2 *NMR Spectroscopy of [K(crypt)][(PN)_2_Ti{O(AlMe_3_)}]*, **3**

**Figure S2:** Room temperature (300 K) ^1^H NMR spectrum of **3** in THF-*d*_8_ referenced to residual solvent peak.^[20]^ Paramagnetic nature of **3** precludes structural assignments, however paramagnetic resonances are marked. Absence of resonances at *ca.* δ -5 are indicative of Ti^III^ with metal-ligand multiple bond character.

3.3 *NMR Spectroscopy of [K(crypt)][(PN)_2_Ti(κ^2^-O_2_C=O)]*, **4**

**Figure S3:** Room temperature (300 K) ^1^H NMR spectrum of **4** in THF-*d*_8_ referenced to residual solvent peak.^[20]^ Paramagnetic nature of **4** precludes structural assignments, however paramagnetic resonances are marked. Resonances at δ -3.47 and -6.22 are indicative of Ti^III^ with no metal-ligand multiple bond character (pseudo halide).

**Figure S4:** Room temperature (300 K) ^1^H NMR spectrum of the reaction of **2** and CO_2_ in THF-*d*_8_ referenced to residual solvent peak.^[20]^ Small amounts of diamagnetic impurities are observed assigned as protio-PN ligand by ^31^P{^1^H} NMR (S3.3.3).

**Figure S5:** Room temperature (300 K) ^31^P{^1^H} NMR spectrum of the reaction of **2** and CO_2_ in THF-*d*_8_ referenced to ^1^H NMR spectrum (S3.3.2).^[8]^ Small amounts of diamagnetic impurities are observed and assigned as protio-PN (δ -7.30).

**Figure S6:** Room temperature (300 K) ^1^H NMR spectrum of the reaction of **4** and excess ClSiMe_3_ (δ 0.21) in C_6_D_6_ referenced to residual solvent peak.^[20]^ Other marked resonances align with those reported for [(PN)_2_TiCl].^[3]^

**Figure S7:** Room temperature (300 K) ^1^H NMR spectrum of the reaction of **4-^13^C** and 2 eq ClSiMe_3_ in C_6_D_6_ referenced to residual solvent peak.^[20]^ THF-*d*_8_ added for solubility. Resonance at δ 0.27 assigned to bis(trimethylsilyl)carbonate^[21]^ and other marked resonances align with those reported for [(PN)_2_TiCl] in C_6_D_6_.^[3]^ White-brown precipitate forms in reaction likely containing cryptand as K(crypt)Cl. Inserts show zoomed in look at paramagnetic resonances.

**Figure S8:** Room temperature (300 K) ^29^Si INEPT-NMR spectrum of the reaction of **4-^13^C** and 2 eq ClSiMe_3_ in C_6_D_6_/THF-*d*_8_ mixture referenced to ^1^H NMR spectrum (S3.3.7).^[8]^ Resonance at δ 30.9 assigned to bis(trimethylsilyl)carbonate.^[22]^

**Figure S9:** Room temperature (300 K) ^13^C{^1^H} NMR spectrum of the reaction of **4-^13^C** and 2 eq ClSiMe_3_ in C_6_D_6_/THF-*d*_8_ mixture referenced to residual solvent peak of C_6_D_6_.^[20]^ Resonance at δ 151.4 and 2.7 assigned to bis(trimethylsilyl)carbonate.^[22-23]^ Resonance at δ 125.4 assigned to ^13^CO_2_.^[24]^

**Figure S10:** Room temperature (300 K) ^1^H ^13^C{^1^H} HSQC NMR spectrum of the reaction of **4-^13^C** and 2 eq ClSiMe_3_ in C_6_D_6_/THF-*d*_8_ mixture referenced to residual solvent peak of C_6_D_6_.^[20]^ Resonance at δ 151.4 and 2.7 assigned to bis(trimethylsilyl)carbonate by cross peak.^[22-23]^

**Figure S11:** Room temperature (300 K) ^1^H NMR spectrum of the reaction of **4** and 1,2-diiodoethane in THF-*d*_8_ referenced to residual solvent peak.^[20]^ Resonance at δ 5.36 assigned to ethylene.^[20]^ Inserts depicts full spectrum enlarged showing no evidence of [(PN)_2_TiI].

**Figure S12:** Room temperature (300 K) ^1^H NMR spectrum of the heating reaction of **4** at 85 °C under vacuum in THF-*d*_8_ referenced to residual solvent peak for 0, 6, and 12 h respectively.^[20]^ Inserts depict zoomed in sections showing loss of paramagnetic resonances.

**Figure S13:** Room temperature (300 K) ^31^P{^1^H} NMR spectrum of the heating reaction of **4** at 85 °C for 12 h under vacuum in THF-*d*_8_ referenced to ^1^H NMR spectrum (S3.3.10).^[8]^


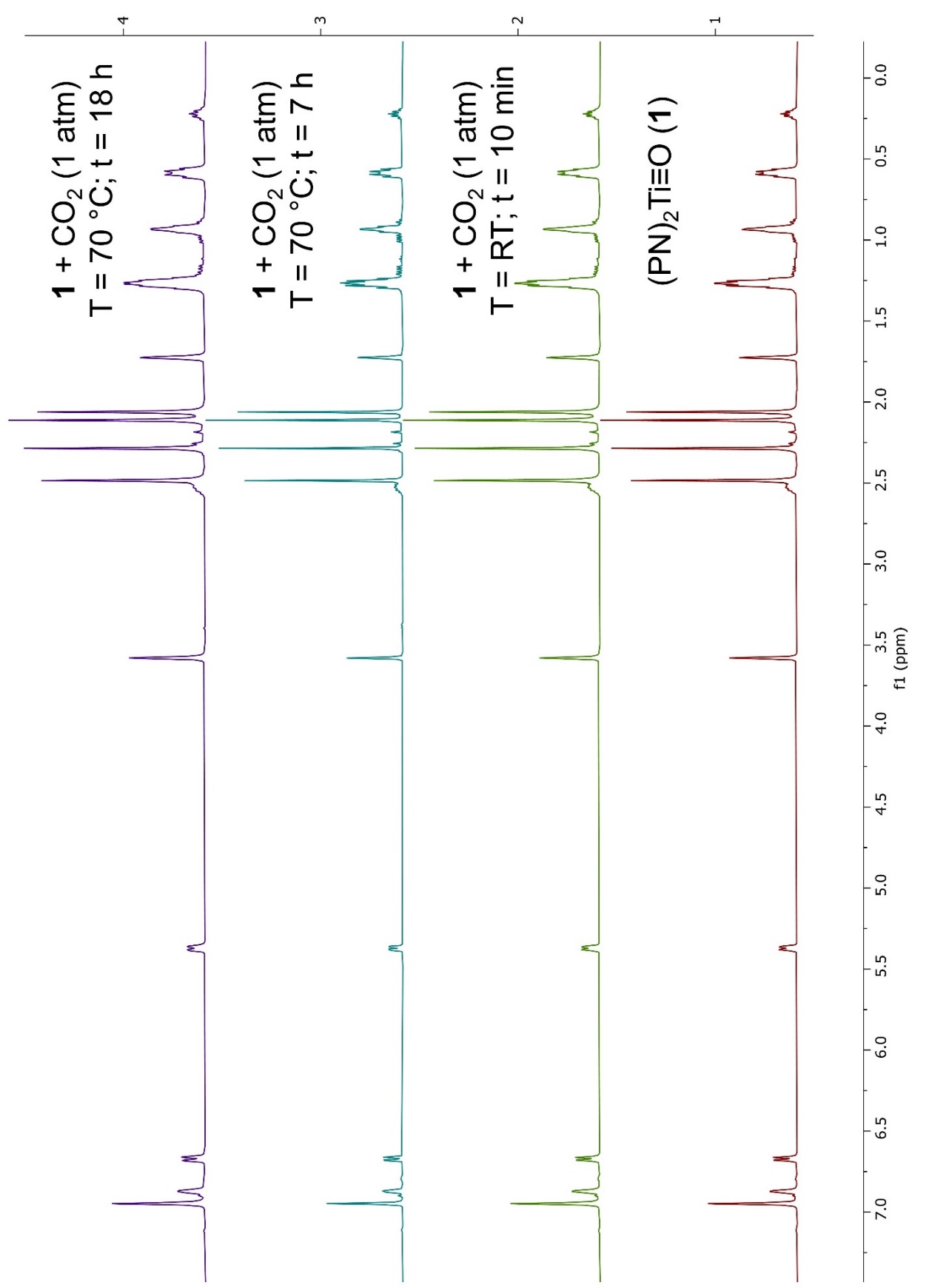


**Figure S14:** Room temperature (300 K) ^1^H NMR spectrum of the heating reaction of **1** + CO_2_ at 70 °C in THF-*d*_8_ referenced to residual solvent peak.^[20]^ No change is observed over 18 h.


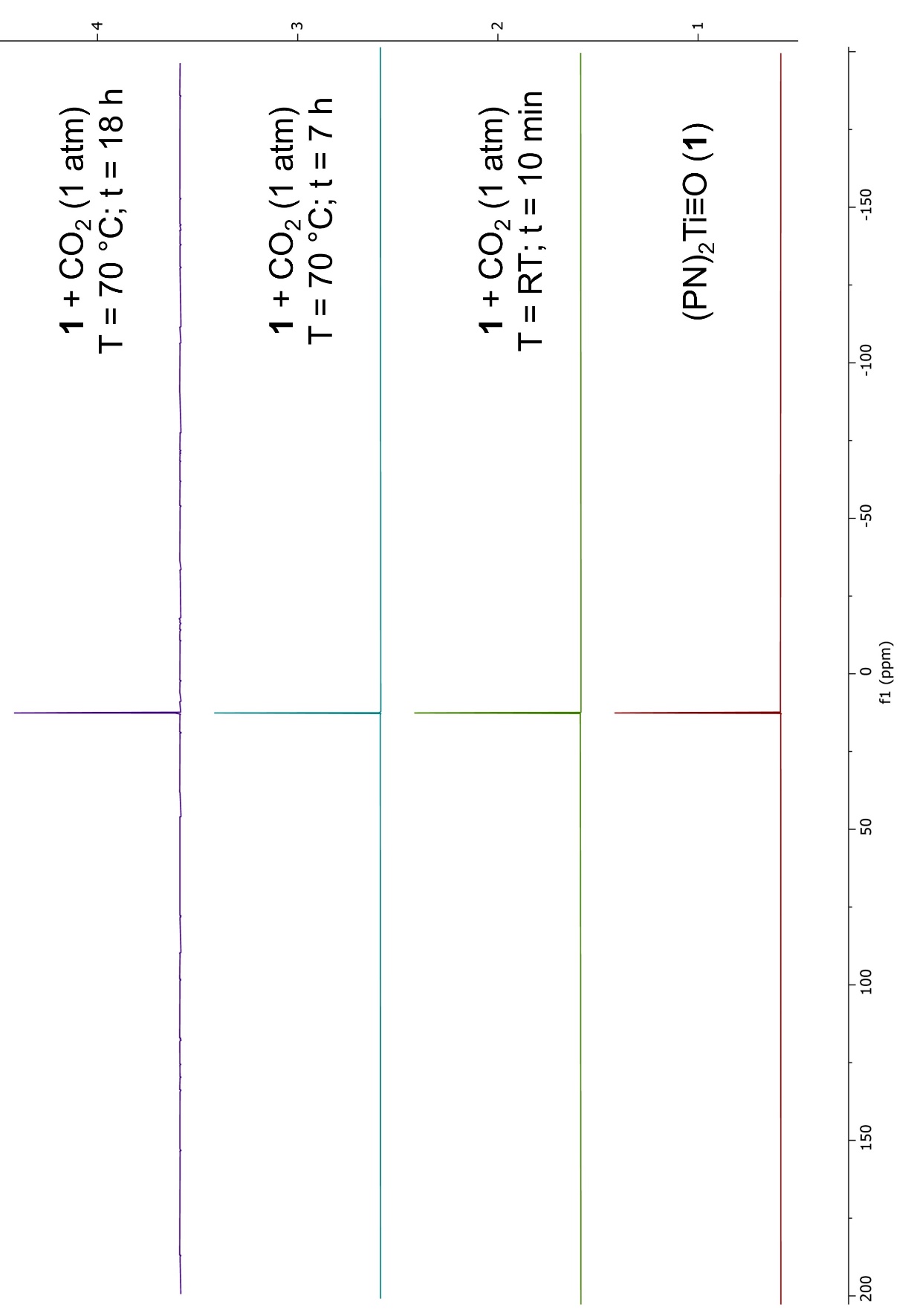
**Figure S15:** Room temperature (300 K) ^31^P{^1^H} NMR spectrum of the heating reaction of **1** + CO_2_ at 70 °C for 18 h in THF-*d*_8_ referenced to ^1^H NMR spectrum (S3.3.12).^[8]^

**4. IR Spectroscopy**

4.1 *IR Spectroscopy of [K(crypt)][(PN)_2_Ti=O]*, **2**

**Figure S16:** IR spectroscopy of **2** (solid state, KBr).

4.2 *IR Spectroscopy of [K(crypt)][(PN)_2_Ti(κ^2^-O_2_C=O)]*, **4**

**Figure S17:** IR spectroscopy of **4** (solid state, KBr).

**Figure S18:** IR spectroscopy of **4-^13^C** (solid state, KBr).

**Figure S19:** Overlay of IR spectra of **4** (orange) and **4-^13^C** (blue) (solid state, KBr).

**Figure S20:** Zoomed in overlay of IR spectra of **4** (orange) and **4-^13^C** (blue) (solid state, KBr).

**5. UV-Vis Spectroscopy**

5.1 *UV-Vis Spectroscopy of [K(crypt)][(PN)_2_Ti=O]*, **2**

**Figure S21:** UV-Vis absorption spectroscopy of **2** in THF full spectrum. Concentrations are listed on the right (mM). Assignments (nm (ε M^-1^cm^-1^)): 215 (71614), 249 (19909), 319 (13657), 398 (3190), 563 (785). Grating changeover occurs at 800 nm.

**Figure S22:** UV-Vis absorption spectroscopy of **2** in THF zoomed in for d-d transition. Concentrations are listed on the right (mM). Assignments (nm (ε M^-1^cm^-1^)): 556 (1119), 682 (253), 944 (135). Grating changeover occurs at 800 nm.

**Figure S23:** UV-Vis absorption spectroscopy of **2** in THF zoomed into 605 nm to 220 nm. Concentrations are listed on the right (mM). Assignments (nm (ε M^-1^cm^-1^)): 249 (19909), 284 (3473), 319 (13657), 398 (3190), 563 (785). Grating changeover occurs at 800 nm.

5.2 *UV-Vis Spectroscopy of [K(crypt)][(PN)_2_Ti{O(AlMe_3_)}]*, **3**

**Figure S24:** UV-Vis absorption spectroscopy of **3** in THF full spectrum. Concentrations are listed on the right (mM). Assignments (nm (ε M^-1^cm^-1^)): 217 (40945), 254 (12063), 273 (10932), 325 (10336), 388 (1780). Grating changeover occurs at 800 nm.

**Figure S25:** UV-Vis absorption spectroscopy of **3** in THF zoomed in for d-d transition. Concentrations are listed on the right (mM). Assignments (nm (ε M^-1^cm^-1^)): 493 (125), 654 (92), 815 (43). Grating changeover occurs at 800 nm.

**Figure S26:** UV-Vis absorption spectroscopy of **3** in THF zoomed into 412 nm to 212 nm. Concentrations are listed on the right (mM). Assignments (nm (ε M^-1^cm^-1^)): 217 (40945), 254 (12063), 273 (10932), 325 (10336), 388 (1780).

5.3 *UV-Vis Spectroscopy of [K(crypt)][(PN)_2_Ti(κ^2^-O_2_C=O)]*, **4**

**Figure S27:** UV-Vis absorption spectroscopy of **4** in THF full spectrum. Concentrations are listed on the right (mM). Assignments (nm (ε M^-1^cm^-1^)): 252 (20949), 280 (14527), 323 (12152), 388 (3381). Grating changeover occurs at 800 nm.

**Figure S28:** UV-Vis absorption spectroscopy of **4** in THF zoomed in for d-d transition. Concentrations are listed on the right (mM). Assignments (nm (ε M^-1^cm^-1^)): 755 (38). Grating changeover occurs at 800 nm.

**Figure S29:** UV-Vis absorption spectroscopy of **4** in THF zoomed into 600 nm to 240 nm. Concentrations are listed on the right (mM). Assignments (nm (ε M^-1^cm^-1^)): 252 (20949), 280 (14527), 323 (12152), 388 (3381), 415 (4894). Grating changeover occurs at 800 nm.

**6. EPR Spectroscopy**

Spin Hamiltonian parameters used in EasySpin to fit the 100K CW X-band EPR of **4**. Errors are obtained from the 95% confidence interval of the fit.

| g_1_ | 1.9641±0.0002 |
| --- | --- |
| g_2_ | 1.9886±0.0003 |
| g_3_ | 1.8968±0.001 |
| ^31^P_1_ A_1_ (MHz) | 63±22 |
| ^31^P_1_ A_2_ (MHz) | 50±2 |
| ^31^P_1_ A_3_ (MHz) | 74±43 |
| ^31^P_2_ A_1_ (MHz) | 64±23 |
| ^31^P_2_ A_2_ (MHz) | 68±2 |
| ^31^P_2_ A_3_ (MHz) | 78±42 |
| sg_1_ | 0.0007±0.005 |
| sg_2_ | 0.0008±0.005 |
| sg_3_ | 0.0102±0.002 |
|  |  |
|  |  |
|  |  |
|  |  |
|  |  |
|  |  |

Spin Hamiltonian parameters for **4** were calculated via density functional theory (DFT) as implemented in ORCA 6.0.^[25]^ The crystallographic coordinates for **4** were used as the input geometry. Calculations used the B3LYP hybrid density functional^[26]^ with the CP(PPP) basis set^[27]^ on Ti and ZORA-def2-TZVP(-f) basis set^[28]^ on all other atoms. The D4 correction was included for dispersion effects.^[29]^ Relativistic effects were treated via ZORA,^[30]^ and solvation was modeled via CPCM in an infinite dielectric.^[31]^ Calculations were accelerated using the RIJCOSX approximation,^[32]^ with the AUTOAUX procedure^[33]^ used to generate fitting basis sets. DFT-calculated Spin Hamiltonian parameters for **4**.

| g_x_ | 1.945 |
| --- | --- |
| g_y_ | 1.964 |
| g_z_ | 1.982 |
| ^31^P_1_ A_x_ (MHz) | –45 |
| ^31^P_1_ A_y_ (MHz) | –40 |
| ^31^P_1_ A_z_ (MHz) | –39 |
| ^31^P_2_ A_x_ (MHz) | –57 |
| ^31^P_2_ A_y_ (MHz) | –49 |
| ^31^P_2_ A_z_ (MHz) | –49 |

**7. Electrochemistry of (2) and (4)**

7.1 *Cyclic voltammogram of* ***2***

**
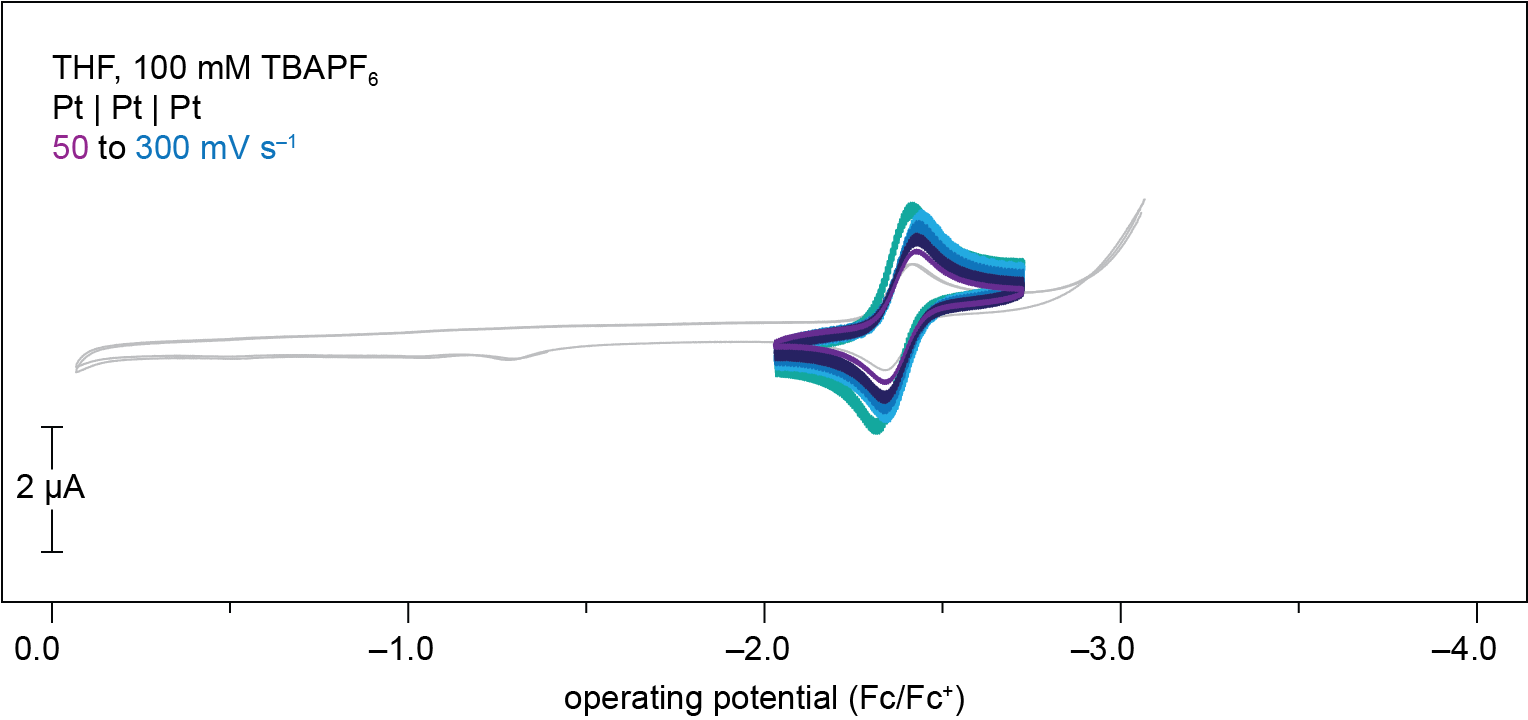
**

**Figure S30.** Cyclic voltammograms of (**2**) in THF at a scan rate of 50 mV s^–1^ to 300 mV s^–1^ with 100 mM TBAPF_6_ as a supporting electrolyte and a Pt working electrode. Overlayed in gray is a CV of (**2**) in a wider window in THF at a scan rate of 50 mV s^–1^ in the same conditions. Scans begin at the most negative potential with the first scan omitted.

7.1 *Cyclic voltammogram of* ***4***

**
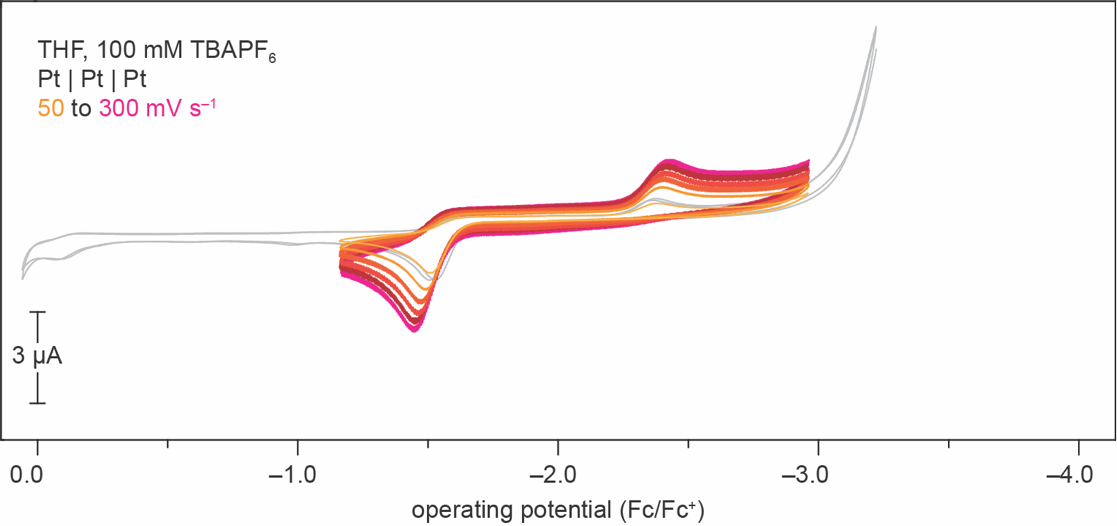
**

**Figure S31.** Cyclic voltammograms of (**4**) in THF at a scan rate of 50 mV s^–1^ to 300 mV s^–1^ with 100 mM TBAPF_6_ as a supporting electrolyte and a Pt working electrode. Overlayed in gray is a CV of (**4**) in a wider window in THF at a scan rate of 50 mV s^–1^ in the same conditions. Scans begin at the most negative potential with the first scan omitted.

1. **X-ray Diffraction Studies**

**Table S1**

| **Compound** | **[K(crypt)][(PN)_2_Ti=O], 2**  **(+ 2 THF)** | **[K(crypt)][(PN)_2_Ti{O(AlMe_3_)}], 3**  **(+ 1.25 THF, 0.75 Hexanes)** | **[K(crypt)][(PN)_2_Ti(**κ*^2^-***O_2_C=O)], 4**  **(+ Et_2_O, Toluene)** |
| --- | --- | --- | --- |
| **CCDC Deposit Number** | **2451583** | **2451584** | **2451585** |
| Empirical formula | C_70_H_114_KN_4_O_9_P_2_Ti | C_74.5_H_127.5_AlKN_4_O_8.25_P_2_Ti | C_67.75_H_107.5_KN_4_O_9.75_P_2_Ti |
| Formula weight | 1304.59 | 1387.22 | 1283.01 |
| Temperature/K | 100 | 100 | 100 |
| Crystal system | monoclinic | monoclinic | orthorhombic |
| Space group | P2_1_/c | P2_1_/n | Pca2_1_ |
| a (Å) | 25.2967(4) | 12.9615(4) | 18.85680(10)Å |
| b (Å) | 12.66636(15) | 25.1645(6) | 31.4188(2)Å |
| c (Å) | 25.1719(4) | 25.0121(10) | 23.84220(10)Å |
| α (°) | 90 | 90 | 90° |
| β (°) | 116.356(2) | 100.067(3) | 90° |
| γ (°) | 90 | 90 | 90° |
| Volume (Å^3^) | 7227.1(2) | 8032.6(5) | 14125.50(13)Å^3^ |
| Z | 4 | 4 | 8 |
| d_calc_ (g/cm^3^) | 1.199 | 1.147 | 1.207 g/cm^3^ |
| μ (mm^‑1^) | 2.372 | 0.261 | 2.428 mm^‑1^ |
| F(000) | 2820.0 | 3010.0 | 5528.0 |
| Crystal size, mm | 0.2 × 0.2 × 0.04 | 0.41 × 0.25 × 0.1 | 0.225 × 0.116 × 0.103 |
| 2θ range for data collection (°) | 7.036 - 149.006 | 4.478 - 50.698 | 5.466 - 149.006° |
| Index ranges | -30 ≤ *h* ≤ 31, -15 ≤ *k* ≤ 11, -30 ≤ *l* ≤ 31 | -15 ≤ *h* ≤ 15, -30 ≤ *k* ≤ 30, -30 ≤ *l* ≤ 29 | -16 ≤ *h* ≤ 15, -17 ≤ *k* ≤ 17, -22 ≤ *l* ≤ 22 |
| Reflections collected | 92836 | 73769 | 291608 |
| Independent reflections | 14747[R(int) = 0.1280] | 14678[R(int) = 0.0593] | 27394[R(int) = 0.0925] |
| Data/restraints/parameters | 14747/174/842 | 14678/503/947 | 27394/223/1627 |
| Goodness-of-fit on F^2^ | 1.041 | 1.134 | 1.037 |
| Final R indexes [I>=2σ (I)] | R_1_ = 0.0690, wR_2_ = 0.1748 | R_1_ = 0.0897, wR_2_ = 0.2200 | R_1_ = 0.0484, wR_2_ = 0.1243 |
| Final R indexes [all data] | R_1_ = 0.0856, wR_2_ = 0.1951 | R_1_ = 0.1081, wR_2_ = 0.2310 | R_1_ = 0.0567, wR_2_ = 0.1317 |
| Largest diff. peak/hole (eÅ^-3^) | 0.90/-1.01 | 1.27/-1.02 | 1.39/-0.97 eÅ^-3^ |

**7.2 Comparison of Structures of (1) and (2)**.

**Figure S32.** Overlay of truncated ORTEP-III 50 % probability thermal ellipsoid plots of **1** and **2** showing the flexion of the PN ligand upon reduction utilizing structural parameters.

**9. References**

[1] N. A. Jones, S. T. Liddle, C. Wilson, P. L. Arnold, *Organometallics* **2007**, *26*, 755-757.

[2] B. L. Tran, M. Pink, D. J. Mindiola, *Organometallics* **2009**, *28*, 2234-2243.

[3] M. E. Carroll, B. Pinter, P. J. Carroll, D. J. Mindiola, *J. Am. Chem. Soc.* **2015**, *137*, 8884-8887.

[4] M. Bhunia, J. S. Mohar, C. Sandoval-Pauker, D. Fehn, E. S. Yang, M. Gau, J. Goicoechea, A. Ozarowski, J. Krzystek, J. Telser, K. Meyer, D. J. Mindiola, *J. Am. Chem. Soc.* **2024**, *146*, 3609-3614.

[5] J. S. Mohar, M. Bhunia, A. L. Laughlin, A. Ozarowski, J. Krzystek, T. M. Keller, M. R. Gau, K. M. Lancaster, J. Telser, D. J. Mindiola, *J. Am. Chem. Soc.* **2025**, *147*, 11625-11631.

[6] L. N. Grant, B. Pinter, T. Kurogi, M. E. Carroll, G. Wu, B. C. Manor, P. J. Carroll, D. J. Mindiola, *Chem. Sci.* **2017**, *8*, 1209-1224.

[7] S. Chakraborty, J. Chattopadhyay, W. Guo, W. E. Billups, *Angew. Chem. Int. Ed.* **2007**, *46*, 4486-4488.

[8] (a) R. K. Harris, E. D. Becker, S. M. C. d. Menezes, R. Goodfellow, P. Granger, *Pure and Appl. Chem.* **2001**, *73*, 1795-1818; (b) R. K. Harris, E. D. Becker, S. M. C. d. Menezes, P. Granger, R. E. Hoffman, K. W. Zilm, *Pure and Appl. Chem.* **2008**, *80*, 59-84.

[9] (a) S. K. Sur, *J. Magn. Resonance (1969)* **1989**, *82*, 169-173; (b) D. F. Evans, *J. Chem. Soc. (Resumed)* **1959**, 2003-2005.

[10] R. Drago, in Physical Methods for Chemists, Saunders College Publishing, 2 edn., 1992, ch. 11, pp. 411-434.

[11] G. A. Bain, J. F. Berry, *J. Chem. Ed.* **2008**, *85*, 532.

[12] S. E. Shaner, K. L. Stone, *J. Chem. Ed.* **2023**, *100*, 2347-2352.

[13] S. Stoll, A. Schweiger, *J. Magn. Reson.* **2006**, *178*, 42-55.

[14] CrysAlisPro 1.171.41.122a: Rigaku Oxford Diffraction, 2021, Rigaku Corporation, Oxford, UK.

[15] CrysAlisPro 1.171.41.122a: Rigaku Oxford Diffraction, 2021, Rigaku Corporation, Oxford, UK.

[16] SCALE3 ABSPACK v1.0.7: an Oxford Diffraction program, 2005, Oxford Diffraction Ltd: Abingdon, UK.

[17] SHELXT v2018/2: Sheldrick, G.M., *Acta Cryst., A,* **2015**, *71*, 3-8.

[18] Sheldrick, G.M., SHELXL-2019/3, *Acta Cryst., A*, **2015**, *71*, 3-8.

[19] Dolomanov, O.V., Bourhis, L.J., Gildea, R.J., Howard, J.A.K., Puschmann, H., Olex2, *J. Appl. Cryst.*, **2009**, *42*, 339-341.

[20] G. R. Fulmer, A. J. M. Miller, N. H. Sherden, H. E. Gottlieb, A. Nudelman, B. M. Stoltz, J. E. Bercaw, K. I. Goldberg, *Organometallics* **2010**, *29*, 2176-2179.

[21] P. Waldschmidt, C. J. Hoerger, J. Riedhammer, F. W. Heinemann, C. T. Hauser, K. Meyer, *Organometallics* **2020**, *39*, 1602-1611.

[22] R. Minkwitz, S. Schneider, *Z. Naturforsch. B* **1998**, *53*, 849-852.

[23] J. S. Silvia, C. C. Cummins, *Chem. Sci.* **2011**, *2*, 1474-1479.

[24] J. P. Krogman, M. W. Bezpalko, B. M. Foxman, C. M. Thomas, *Inorg. Chem.* **2013**, *52*, 3022-3031.

[25] (a) F. Neese, *WIREs Computational Molecular Science* **2022**, *12*; (b) F. Neese, *J. Chem. Phys.* **2001**, *115*, 11080-11096; (c) F. Neese, *J. Chem. Phys.***2003**, *118*, 3939-3948; (d) F. Neese, *J. Chem. Phys.* **2005**, *122*, 34107.

[26] (a) A. D. Becke, *J. Chem. Phys.* **1993**, *98*, 5648-5652; (b) P. J. Stephens, F. J. Devlin, C. F. Chabalowski, M. J. Frisch, *J. Phys. Chem.* **1994**, *98*, 11623-11627.

[27] F. Neese, *Inorganica Chimica Acta* **2002**, *337*, 181-192.

[28] F. Weigend, R. Ahlrichs, *Phys Chem Chem Phys* **2005**, *7*, 3297-3305.

[29] E. Caldeweyher, J. M. Mewes, S. Ehlert, S. Grimme, *Phys Chem Chem Phys* **2020**, *22*, 8499-8512.

[30] C. van Wüllen, *The Journal of Chemical Physics* **1998**, *109*, 392-399.

[31] Y. Takano, K. N. Houk, *J. Chemical Theory and Computation* **2005**, *1*, 70-77.

[32] F. Neese, F. Wennmohs, A. Hansen, U. Becker, *Chem. Phys.* **2009**, *356*, 98-109.

[33] G. L. Stoychev, A. A. Auer, F. Neese, *J Chem Theory Comput* **2017**, *13*, 554-562.
